# Biomechanical and clinical evaluation of minimal invasive plate osteosynthesis for two-part clavicle shaft fractures

**DOI:** 10.1186/s12891-023-06699-x

**Published:** 2023-07-25

**Authors:** Antonia Schlüßler, Manuel Fehrenbacher, Richard Frank Richter, Eric Tille, Achim Biewener, Jörg Nowotny

**Affiliations:** 1 University Centre for Orthopaedic, Trauma- and Plastic Surgery (OUPC), University Hospital Carl Gustav Carus, Technical University Dresden, Fetscherstraße 74, 01307 Dresden, Germany; 2 Centre for Translational Bone, Joint and Soft Tissue Research, Technical University Dresden, Dresden, Germany

**Keywords:** Clavicle fracture, Shaft fracture, Osteosynthesis, Biomechanical testing, LCP, Locking screw

## Abstract

**Background:**

Many surgical treatment methods exist for clavicle shaft fractures. A locking compression plate (LCP) fixation with three screws per fracture side is commonly used. For certain fractures a stabilization with 2 screws per side is potentially suitable, offering the advantage of reduced soft tissue approach, while avoiding the disadvantages of minimally-invasive nailing at the same time. This hypothesis was evaluated biomechanically and clinically.

**Methods:**

Four treatment procedures were investigated biomechanically using composite human clavicle specimens. A load-to-failure test was performed using a three-point cantilever test. In group 1, a simple shaft fracture was simulated and stabilized with 2 screws per fracture side (5-hole LCP). In the second group 3 screws per side (7-hole LCP) were used. In group 3, a non-reduced fracture zone was simulated and treated with 3 screws per side (7-hole LCP). In group 4, an anatomically reduced fracture zone was simulated and treated with 3 screws per side (7-hole LCP). Furthermore 27 patients treated with a short plate and 2 screws per side (similar to group 1) were assessed after a minimum follow-up of 12 months (Constant and DASH Score).

**Results:**

The maximum load-to-failure of group 1 was 367N. We observed the highest load-to-failure in group 2 with 497N and the lowest in group 3 with 90N. In group 4 a maximum load-to-failure of 298N could be evaluated. There was no significant difference in load-to-failure between the treatment of a simple clavicle fracture using 5- or 7-hole LCP (*p* = 0.121). However, we found a significant difference of load-to-failure between the simple and anatomically reduced fracture using a 7-hole plate (*p* = 0.014). The mean constant score of the surgically treated patients was 95 and the DASH score 3.0. Fracture consolidation was observed in 96.3%.

**Conclusions:**

For certain non-fragmented and well interlocking 2-part fractures, a plate osteosynthesis fixed with only 2 screws per fracture side might offer sufficient biomechanical stability, better soft tissue preservation and comparable fusion rates compared to the operative treatment with 3 screws per side. However, the maximum load-to-failure of the 7-hole LCP was higher than of the 5-hole LCP, but this difference was not statistically significant.

**Trial registration:**

Approval from the ethics committee of the Technical University of Dresden was retrospectively obtained (EK 588122019).

**Supplementary Information:**

The online version contains supplementary material available at 10.1186/s12891-023-06699-x.

## Background

With a prevalence of about 2.6–10% clavicle fractures are one of the most common fractures of the shoulder girdle [1, 2]. Most fractures affect the middle third of the shaft with about 76%, followed by the lateral third with 19% and the medial third with about 4% [3]. The need for anatomical reconstruction (avoiding shortening of the bone) is caused by the function of the clavicle as the only osseous connection between the shoulder girdle and the torso. Accordingly, it plays an essential role in the rotational movements between the AC and SC joints during all shoulder joint movements.

Younger male patients up to the age of 30 years who have had an accident involving high impact and direct fall or force on the involved shoulder are most commonly affected [4]. In the treatment of clavicle fractures, the basic goal is to avoid shortening, as this can result in reduced range of movement of the upper extremity and persistent pain in the shoulder girdle area [5]. With good to very good results, the conservative treatment remains the treatment of choice for non-displaced or minimally displaced fractures [3, 6]. In recent years, studies have analysed the extent of fracture dislocation as a major risk factor for pseudarthrosis (up to 15% in the case of dislocated fractures) [6, 7]. Thus, the indications for surgical therapy are dislocated fractures (more than one shaft width or 100%) and fractures with significant shortening (> 14 mm in women and 18 mm in men) [5, 7]. The surgical standard procedure consists of open reduction and plate osteosynthesis and shows low complication rates (approx. 5%), good to very good clinical results and high fusion rates (> 95%) [8–10]. There is an ongoing debate, however about the optimal fixation technique, since there are many possibilities why a plate osteosynthesis can fail (e.g. screw pullout, plate bending, plate breakage) [11, 12]. Nevertheless, the overall number of surgical procedures has increased in the last years as a therapy that allows early functional treatment of the shoulder joint and girdle [13, 14].

Especially the shaft area of the clavicle is often a cosmetically sensitive region. Thus, the trend in recent years has been leaning towards minimally invasive therapy options. One commonly available treatment is the intramedullary nail osteosynthesis such as the elastic-stable intramedullary nailing (ESIN) [14, 15]. A serious disadvantage of this method compared to locking compression plate (LCP) osteosynthesis is a worse rotational stability, which has been proven biomechanically [16]. Technical problems, especially due to a narrow medullary canal are frequent and can make an intramedullary nail osteosynthesis impossible. In these cases a conversion to an open reduction and plate osteosynthesis must be performed [17].

The present study therefore aimed to evaluate whether a short 3.5 mm LCP fixed with only 4 screws is biomechanically and clinically sufficient for certain 2-part clavicle shaft fractures.

## Methods

### Biomechanical analysis

#### Samples

Since the clavicle fracture is an injury of the young age (generally no presence of osteoporosis) a cadaver test (with mostly osteoporotic specimens) was avoided and biomechanically standardized composite bone was used in order to eliminate the structural variability in human bone [18]. Therefore, composite bones (Clavicle, 4th generation, photonic-crystal fiber, Sawbones, Europe) which mimic the properties of human bones were chosen. For each study group 5 clavicles were biomechanically examined. This has been proven to be an adequate standard in other studies before [19, 20]. A simple transverse fracture (15–2 A3 according to AO classification) was simulated for group 1 and 2, whereby a non-reduced (unstable) multi-fragmentary diaphyseal fracture (group 3) and reduced (stable) multi-fragmentary diaphyseal fracture with a 10 mm intermediary fragment (15–2 C2) were simulated (group 4) in the other two groups. The fracture was placed in the mid-shaft 5.6 cm from the most lateral end of the clavicle with an oscillating saw.

#### Implants

Fixation was realized according to the modern procedures of plate osteosynthesis following the guidelines of the AO Foundation (“Arbeitsgemeinschaft für Osteoynthesefragen”, Swiss) with an LCP in a superior position. Therefore, initially, one non-locking screw per fracture side was drilled decentralized close to the fracture (compression both to the bone and to the fracture gap). Hereafter osteosynthesis was “locked” with one or two bicortical locking screws per fracture side peripherally, depending on the group.

**Fig. 1:**
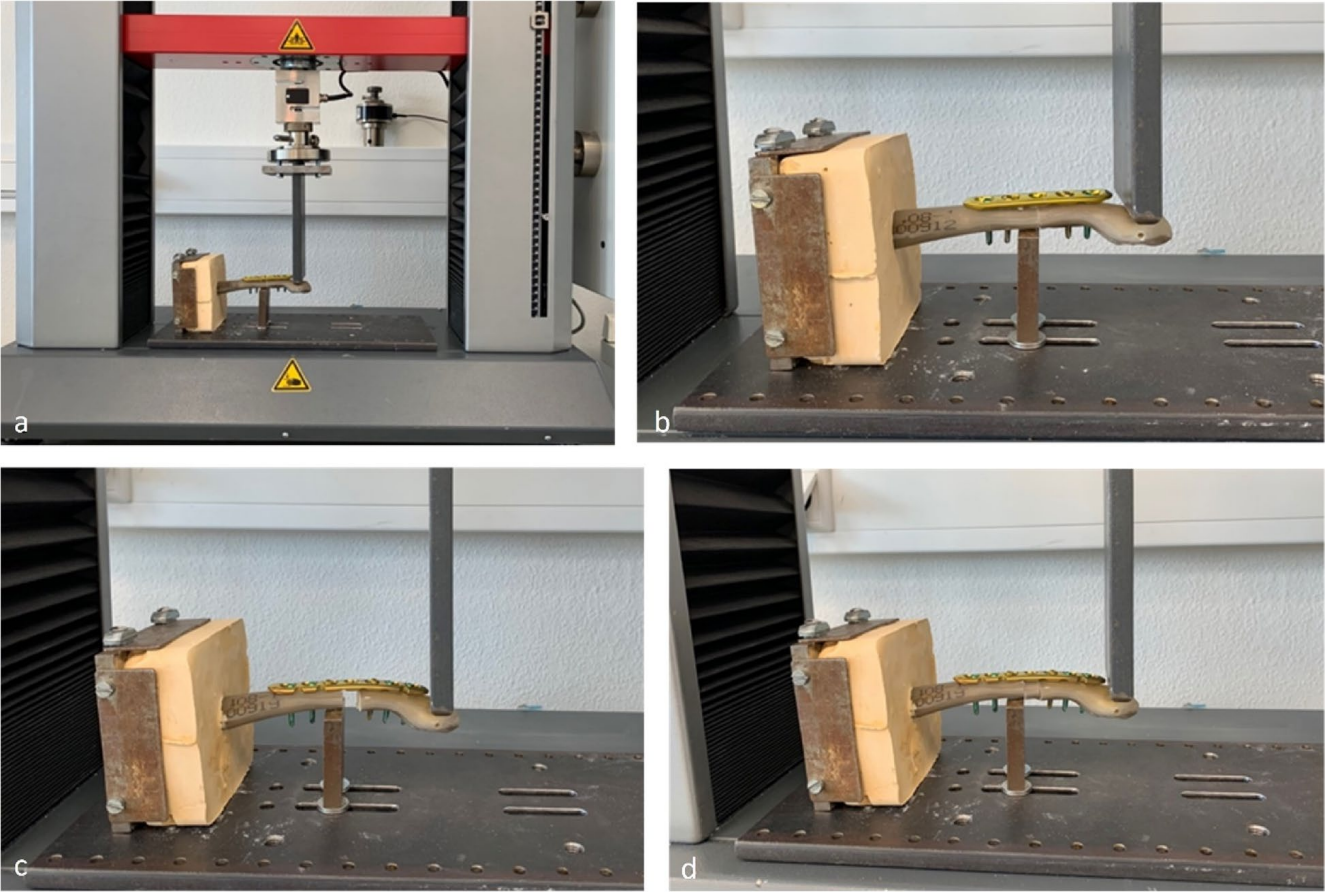
Biomechanical test setup (**a**); **b** group 1: simple fracture with 5-hole LCP; **c** group 3: non-reduced multi-fragmentary diaphyseal fracture (unstable) with 7-hole LCP; **d** group 4: reduced multi-fragmentary diaphyseal fracture (stable) with 7-hole LCP

**Fig. 2:**
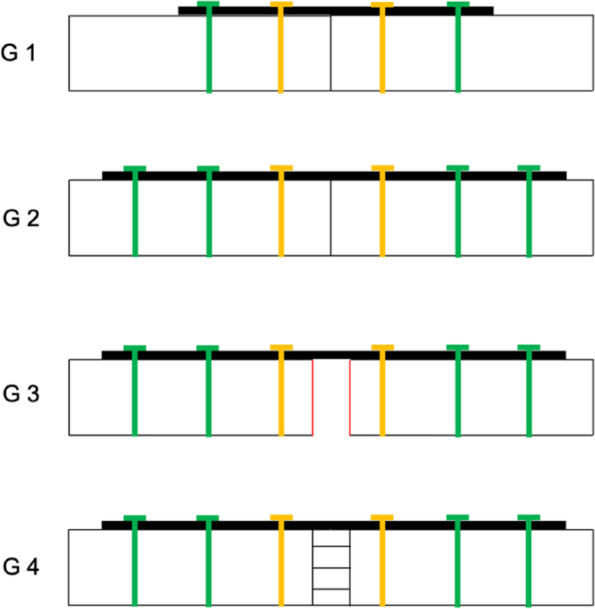
Schematic illustration of the investigated groups. group 1: simple fracture with 5-hole LCP; group 2: simple fracture with 7-hole LCP; group 3: non-reduced multi-fragmentary diaphyseal fracture (unstable) with 7-hole LCP, group 4: reduced multi-fragmentary diaphyseal fracture (stable) with 7-hole LCP. (yellow: non-locking screw, green: locking screw)

The four groups that were established and investigated are shown in Figs. 1 b-d and  2: in group 1 a simple non-fragmented clavicle shaft fracture (15–2 A3 according to AO classification) was simulated and stabilized with 2 screws per fracture side (1 non-locking and 1 locking) and a 5-hole LCP (3.5 mm LCP, Titanium, Synthes, USA). As a direct comparison, in group 2 a simple non-fragmented clavicle shaft fracture was treated with 3 screws per fracture side (1 non-locking and 2 locking) with a 7-hole LCP and evaluated. In group 3 a non-reduced diaphyseal fracture (unstable) was fixed using 3 screws (1 non-locking and 2 locking) per fracture side with a 7-hole LCP (3.5 mm LCP, Titanium, Synthes, USA). Finally, in group 4 a reduced (stable) multi-fragmentary diaphyseal fracture was addressed with 3 screws (1 non-locking and 2 locking) per fracture side also using a 7-hole LCP (3.5 mm LCP, Titanium, Synthes, USA).

#### Test setup

The clavicles were placed orthograde into bone cement (Dental Plaster Type 4, Excalibur, water/plaster ratio 22:100, Siladent, Dr. Böhme & Schöps GmbH, Germany) in an exact 90° position (Fig. 1a). We used a three-point cantilever measurement method in the current study because it is commonly used, provides excellent comparability with other studies and is recognized as a general physiological measurement setup [21–25]. The clavicles within the bone cement block were positioned and fixed with a steel screw /-plate construction under the force bar (Fig. 1a). An abutment was placed underneath the clavicle, medially to the fracture, simulating the fulcrum of the clavicle over the first rib. The force was applied from cranial direction at the lateral end of the clavicle. Uniaxial Compression tests were performed using a Zwick/Roell® series testing system (Z010, Zwick GmbH, Ulm, Germany) equipped with a 10 kN load cell. The tests were performed at 22 °C, 65% relative humidity. At the beginning of the investigation, a repetitive preloading was performed with 250 cycles at 1 Hz between 5 and 75 N, similar to the preloading described by Renfree et al. and Celestre et al. [22, 26]. Thereafter, a load-to-failure compression test was carried out at the same position, with a speed of 10 mm/min. The applied force and distance were measured. Also, the stiffness was measured from the linear part of the load–displacement curve in N/mm.

### Clinical investigation

From 2012 to 2021, 27 patients with suitable fractures that were treated with a short LCP were retrospectively included and examined after a minimum follow-up of 12 months. For the minimal-invasive approach, an approximately 4 cm modified sabercut approach was performed parallel to Langer's lines. After open reduction and optional temporarily K-wire fixation a 4- or 5-hole 3.5 mm locking compression plate (LCP, Titanium, Synthes, USA) or reconstruction plate (Synthes, USA) was inserted by tunnelling of the soft tissue medially and laterally over the fracture through gentle traction with a retractor. Initially the plate was attached by drilling the holes closest to the fracture using the dynamic compression (DC) principle with bicortical non-locking screws for fracture compression. Subsequently, the two peripheral holes were fixed with bicortical locking screws (*n* = 19) or further non-locking screws (*n* = 8). In 4 cases an additional lag screw was used before placing the plate. After documentation of the osteosynthesis under image intensifier (X-ray), the wound was lavaged and closed in several layers.

At an average follow-up of 26 months (min: 12; max: 83) demographic data, rate of bone fusion, the Constant Score (CS) and DASH Score (DS) were evaluated. Fracture consolidation was defined as a radiographic fusion or removal of the plate with proven bone fusion.

Statistical analysis was performed with SPSS Statistics software (version 28; IBM, Armonk, NY, USA) for descriptive statistics. All numerical data are presented as mean with standard deviation as well as the range (minimum—maximum). A nonparametric test for comparison (Kruskal–Wallis Test) was used in terms of their significance level. The significance level was chosen at *p* < 0.05.

## Results

### Biomechanical analysis

**Fig. 3:**
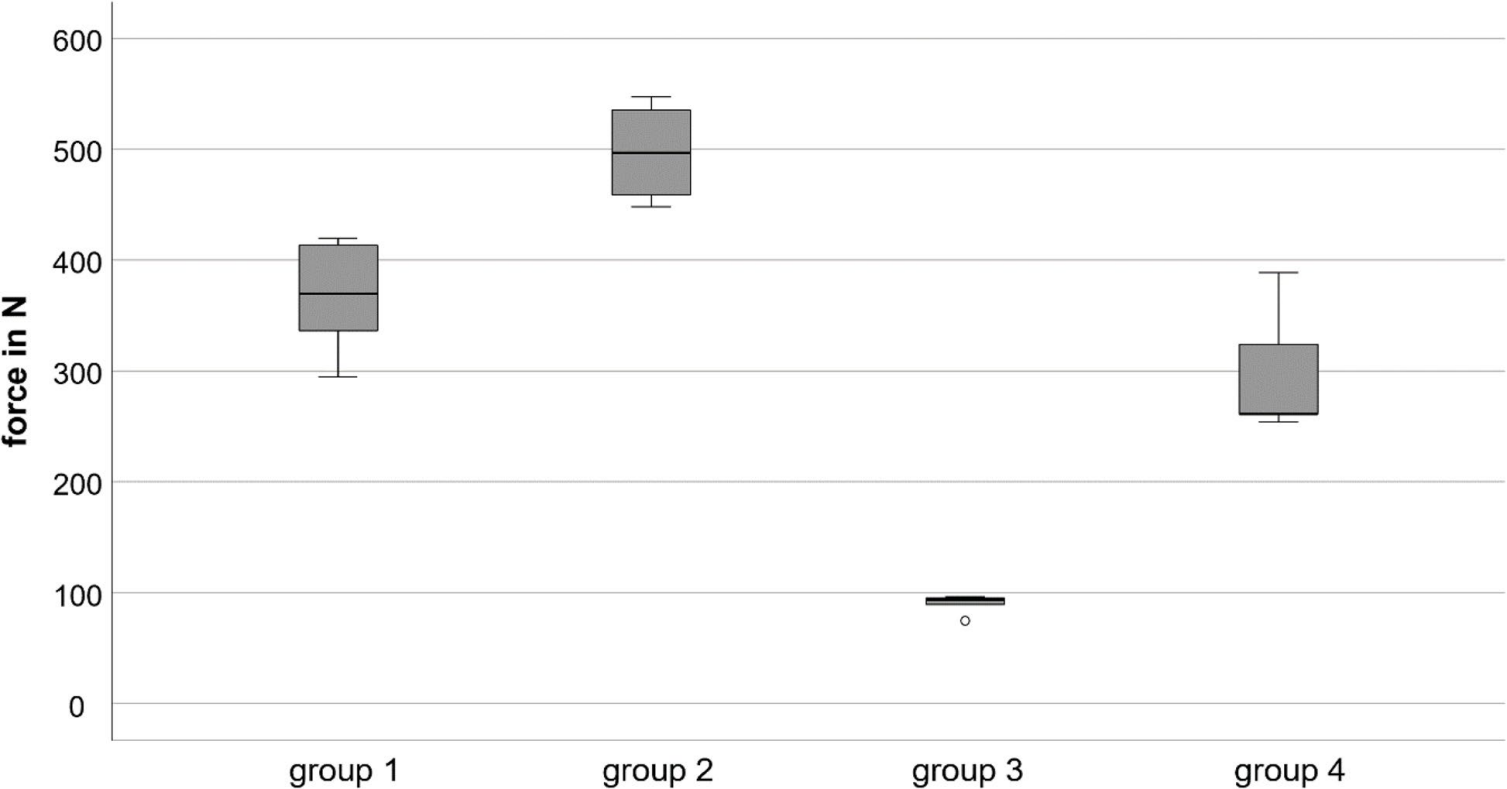
Boxplot of outcome parameter: maximum load-to-failure in N. group 1: simple fracture with 5-hole LCP; group 2: simple fracture with 7-hole LCP; group 3: non-reduced multi-fragmentary diaphyseal fracture (unstable) with 7-hole LCP; group 4: reduced multi-fragmentary diaphyseal fracture (stable) with 7-hole LCP (* = *p* value < 0.05)

Between the four investigated groups, different maximum load-to-failure and stiffness could be evaluated. The maximum load-to-failure of group 1 was 367 N (min: 295, max: 420, SD: 53) respective. No osteosynthesis failure (screw pullout or plate breakage) was seen. In all cases a failure occurred medial to the plate, which can often be observed in cantilever tests. The maximum load-to-failure of the 7-hole LCP in group 2 was highest with 497 N (min: 448, max: 547, SD: 44) and lowest in group 3 with 90 N (min: 75, max: 96, SD: 9). Simulating an anatomically reduced fracture by bridging the fracture gap with a bony insert, a maximum load-to-failure of 298 N (min: 254, max: 389, SD: 58) was evaluated in group 4. There was no significant difference between the load-to-failure within the treatment of a simple clavicle fracture using a 5- or a 7-hole LCP (group 1 vs. group 2: *p* = 0.121). We did however observe a significant difference of load-to-failure between the simple and anatomically reduced fracture using 7-hole plates (group 2 vs. group 4: *p* = 0.014). Especially the non-reduced (unstable) clavicle shaft fracture stabilized with a 7-hole plate (group 3) had a significantly reduced load-to-failure compared to both, the 5-hole (*p* = 0.001) and the reduced (stable) 7-hole osteosynthesis (*p* = 0.014). During the examination of the 7-hole plate, a bending of the plate could always be observed as a failure pattern without any bone fracture or screw pullout (unlike the 5-hole plate). Table 1 and Fig. 3 summarize the data.

**Table 1:**
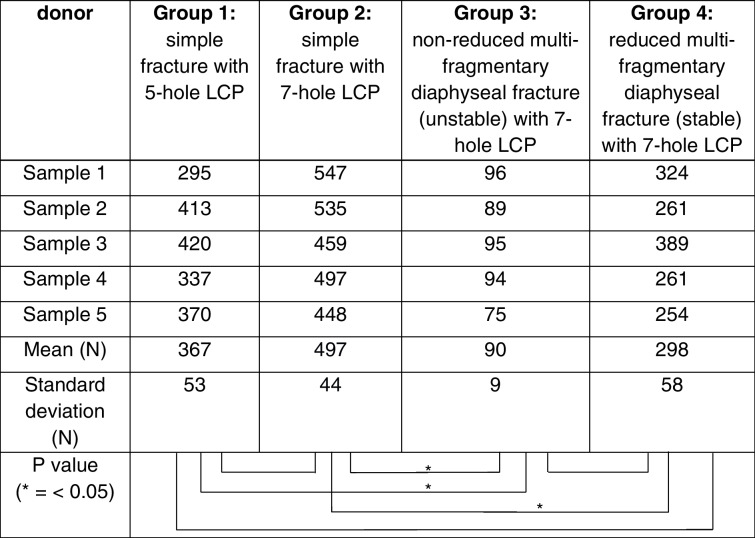
Overview of maximal load-to-failure according to the groups

**Fig. 4:**
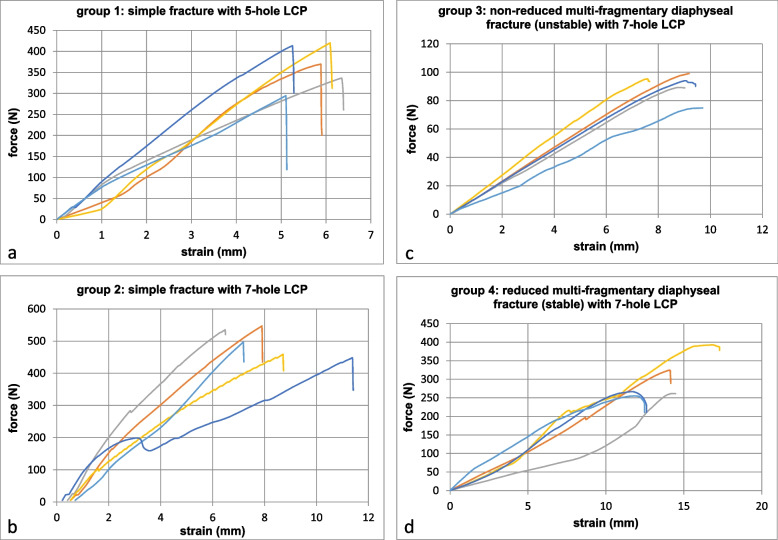
Force–strain curves **a** group 1: simple fracture with 5-hole LCP; **b** group 2: simple fracture with 7-hole LCP; **c** group 3: non-reduced multi-fragmentary diaphyseal fracture (unstable) with 7-hole LCP, **d** group 4: reduced multi-fragmentary diaphyseal fracture (stable) with 7-hole LCP

With regard to the stiffness, differences between the investigated groups could likewise be evaluated, whereby the force-strain curves show an almost linear increase (Fig. 4). As expected, the stiffness was highest for stabilization of the simple fracture (5-hole plate: 66.5 N/mm, min: 51.1, max: 80.8, SD: 13.1 and 7-hole plate: 70.6 N/mm, min: 57.5, max: 78.9, SD: 8.7) and lowest for stabilization of a non-reduced (unstable) fracture fixed with a 7-hole plate (10.8 N/mm, min: 8.4, max: 13.1, SD: 1.7). For the reduced multi-fragmentary diaphyseal fracture (stable), a value of 23.0 N/mm (min: 16.5, max: 26.7, SD: 4.1) was evaluated. Consequently, no significant differences in stiffness were observed comparing group 1 and 2 (*p* = 0.789), but comparing group 3 to 1 (*p* = 0.001) and 2 to 1 (*p* < 0.001). Table 2 summarizes the data.

**Table 2:**
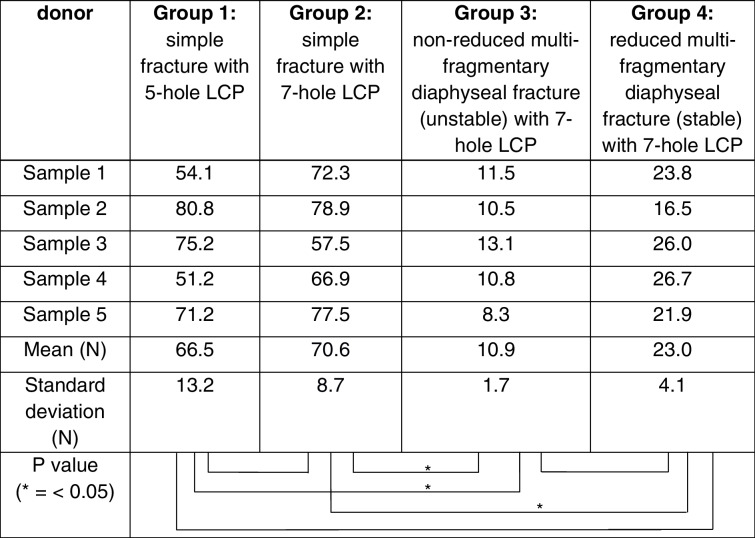
Overview of stiffness according to the groups

### Clinical evaluation

**Fig. 5:**
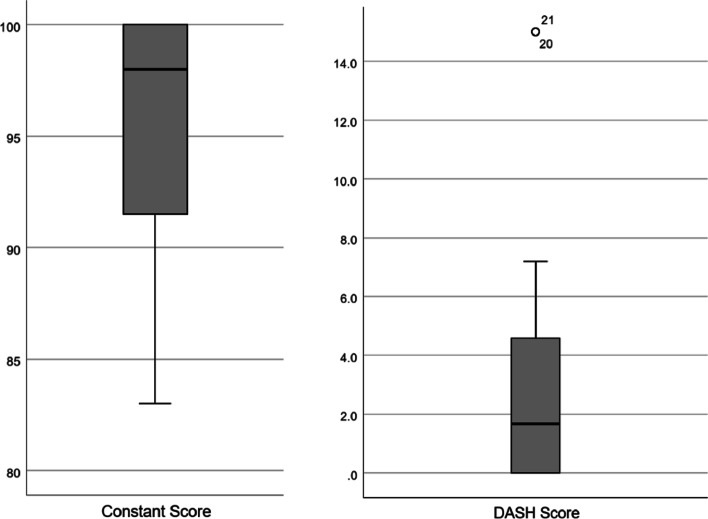
Boxplot of clinical outcome parameters (left) Constant Score 95 (min: 83, max: 100, SD: 5.7); (right) DASH Score 3.0 (min: 0, max: 15.0, SD: 4.3)

The mean patient age was 29 years (min: 15, max: 78, SD: 16.1). 33.3% of the patients were female. The average follow-up period was 26 months (min: 12, max: 83, SD: 16.3). 6 patients (22.2%) were treated with a 4-hole, 21 patients (77.8%) with a 5-hole plate. An additional lag screw was used in 3 cases (11.1%). In 8 patients, only non-locking screws were used for fixation (29.6%). The mean Constant score was 95 (min: 83, max: 100, SD: 5.7) and the DASH score 3.0 (min: 0, max: 15.0, SD: 4.3) (Fig. 5).

**Fig. 6:**
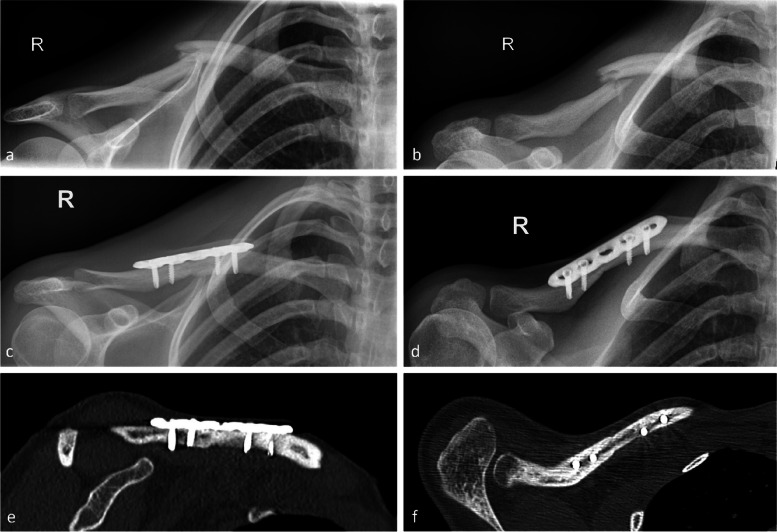
Radiological example of a 2-part clavicle shaft fracture (15.2A according to AO classification) (**a** and **b**) and radiological result 3 years (**c** and **d**) after surgical treatment using a 5-hole LCP. **e** and **f** CT scan showing a full consolidation 21 months after surgery

**Fig. 7:**
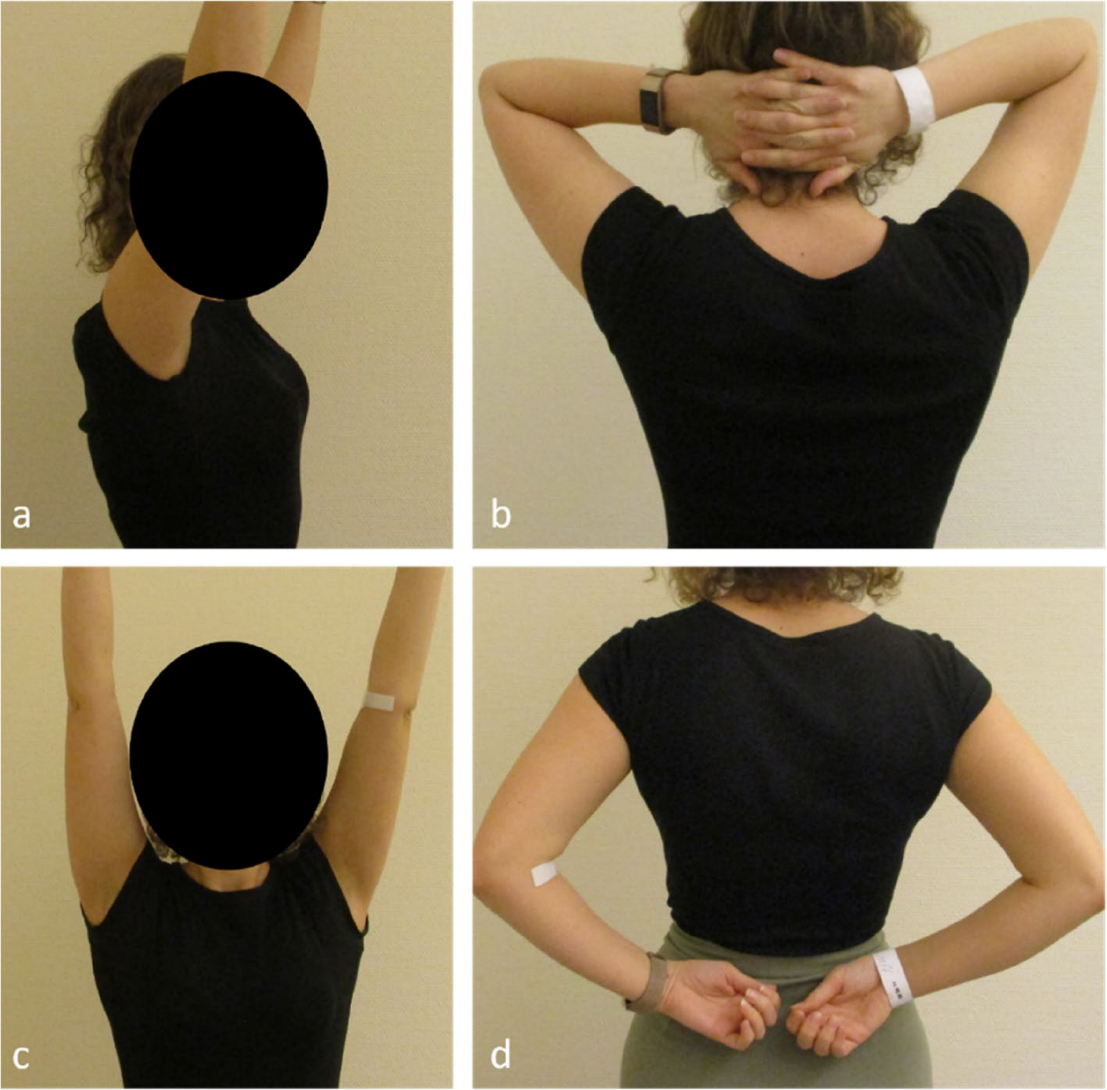
**a**-**d** Clinical example 3 years after surgical treatment with 5-hole LCP on the right clavicle, same patient as in Fig. 6

A consolidation of the fracture was observed in 96.3% (26/27) of the fractures in patients treated with a minimal invasive 4- or 5-hole LCP more than 1 year after the operation. A radiological and clinical example is shown in Figs. 6 and 7. In one case (*n* = 1; 3.7%), an early osteosynthesis failure occurred 6 weeks after 5-hole plate osteosynthesis. In the revision surgery (conversion to a 6-hole plate) the position of the plate was found to be incorrect and the lateral screws were placed just unicortically, which might have happened due to the very slim bone of that young woman. Also, the surgeon used only non-locking screws, which should have been avoided when using only 2 screws on each side of the fracture. Consolidation was achieved after revision surgery. Additional file 1 shows the imaging succession of that case.

In 44% of the cases a material removal has been performed after consolidation of the fracture.

## Discussion

The present study investigated the biomechanical properties as well as the clinical outcome of a shortened locking plate osteosynthesis for simple clavicle shaft fractures. We were able to confirm our initial null hypothesis and show that a well-reduced simple fracture treated with a shortened 5-hole plate shows a sufficient stability. This corresponds to the preliminary expectations, since the two inner cortical screws (DC principle) cause high compression in the fracture line, which is then secured by 1 interlocking screw per fracture side. The stability of an identical fracture treated with a 7-hole plate and an anatomically reduced multi-fragment clavicle fracture, which was also treated with a 7-hole plate, did not differ significantly. Furthermore, the stability of a 5-hole/4-screw plate osteosynthesis for a 2-part fracture was significantly higher than the 7-hole/6-screw plate osteosynthesis of a non-reduced multi-fragmentary diaphyseal fracture.

We decided to use a three-point cantilever test setup, since it is commonly used for this kind of investigation and therefore accepted as a physiological correlate of the load on the shoulder girdle [21–24]. However, human motion is more complex, which might make this model considered unfavorable. Screw pullout, a common clinical complication, cannot be adequately simulated and fractures often occur adjacent to the plate [27]. This was also the finding in our study, where in the most cases a fracture occurred at the medial edge of the plate.

Several studies have investigated the stability of different types of clavicle shaft fracture treatment biomechanically [11, 21, 28–31]. Hulsmans et al. conducted a systematic review of 15 biomechanical studies and found that plate fixation seemed to provide more stability than intramedullary fixation. Also, superior plating showed higher stiffness and strength than anteroinferior plating [32].

Most authors investigated treatment with 3 screws per fracture side. Hamman et al. compared bicortical non-locking screws with unicortical locking screws and saw that unicortical fixation using pre-contoured plates and locking screws has a similar biomechanical profile compared to gold standard non-locking bicortical screws in cyclic axial compression and axial load-to-failure [11]. Bravman et al. evaluated a bicortical and unicortical fixation using 3 locking screws per fracture side in a superior position (3.5 mm LCP) [33]. They found no significant differences between unicortical and bicortical fixation in failure load, cantilever bending, and cross body stiffness. However, bicortical fixation was significantly stiffer than unicortical fixation in torsion for the same plates. Looft et al. compared the bending and torsional strength of a uni-, bicortical locking screw plate construct and a hybrid unicortical plate construct (with central locked and outer non-locked long oblique screws) for clavicle fracture fixation [34]. They also found no significant differences in bending stiffness or ultimate bending moment between all three plating techniques. Modern fixation procedures, as advised by the AO, combine the use of non-locking and locking screws. This has been implemented in the present study to achieve good initial compression of the fracture itself and of the plate to the bone.

There are also several studies that have investigated biomechanical differences between 2- and 3-screw treatments per fracture side. Larsen et al. examined the question by using a 7-hole midshaft clavicle plate (Arthrex) and biomechanically found no significant differences for cyclic displacement, stiffness, yield load or ultimate load between the groups [27]. However, the study used a different experimental setup on one hand and a different type of fixation and implant (3 non-locking vs. 2 locking screws per fracture side; stainless steel implant) on the other hand. The results are therefore not directly comparable. Also, in contrast to the current study, a substantially higher load-to-failure had been measured (2 screws: 2496 ± 1102 N versus 3 screws: 2715 ± 1150 N). Although the absolute values are not comparable, the general conclusion is the same: there is no significant biomechanical difference when using 2 screws versus 3 screws per fracture side in fixation of a clavicle fracture. These conclusions were also proven for long tubular bones. Grawe et al. investigated a synthetic tubular bone model with normal and osteoporotic bone density by using 2 locking screws (5-hole LCP) versus 3 non-locking screws (7-hole LCP) on each side of the fracture gap [19]. They found comparable mechanical performance of locking plate constructs using only 2 locking screw – versus 3 non-locking screw constructs in osteoporotic bone. However, besides the present study, there is no other examination that investigates this issue by using a modern plate osteosynthesis (5-hole LCP) with primary non-locking screws for compression and later fixation with 1 or 2 locking screws, that we know of.

Furthermore, different plate positions were examined. Kontautas et al. evaluated the 3.5 mm locking reconstruction plate in an anteroinferior or a superior position for transverse clavicle fractures and found a significantly greater biomechanical stability with a superior plate osteosynthesis [35]. Celestre et al. confirmed these findings [22]. Since the superior plate position is generally accepted and clinically validated, it was also used in the present study. Nevertheless, other studies have evaluated a slightly higher value for the average load-to-failure (e.g. pullout forces applied parallel to the long axis) [27, 35]. Toogood et al. biomechanically compared a superior and an anterior plate positioning for clinically relevant midshaft clavicle fractures [36]. The results showed different advantages and disadvantages of positioning and therefore could not give a general recommendation for either superior or anterior plate positioning.

In the clinical part of the present study, non-union was not seen in the radiological examination, although in one case (3.7%) an early osteosynthesis failure (6 weeks after surgery) occurred after 5-hole plate osteosynthesis. However, the intra- and postoperative analysis was able to show, that two screws were drilled tangentially to the narrow medullary canal, resulting in an impaired screw retention force. Ranalletta et al. reported 1 non-union in 68 interventions (1.5%) and 1 hardware loosening (1,5%) while using a precontoured locking plate and a minimum of 3 screws on each side of the fracture [10]. Martin et al. found a similar non-union rate (1.3%) for compression plates in their systematic review and meta-analysis, which can be considered comparable overall [37].

A commonly seen complication is skin numbness around the surgical scar due to an injury of the cutaneous supraclavicular nerve branches. Beirer et al. have shown that a smaller skin incision reduces the area of chest wall numbness [17]. The mini-open plating method (“MIPO”) showed an average skin incision length that was 32.5 mm shorter than in conventional plating (61.3 ± 12.3 mm vs. 93.8 ± 17.7 mm) and the area of anterior chest wall numbness could therefore be reduced from 19.8 ± 17.0 cm^2^ to 4.7 ± 3.4 cm^2^ (*p* < 0,05). With the technique that was used in the present study, we were able to reduce the length of the incision in order to reduce skin numbness and other complications.

The clinical results are very good and comparable with other clinical examinations. Ranalletta et al. evaluated displaced midshaft clavicular fractures treated with precontoured locking plates and found a Constant Score of 97.8 and a DASH of 1.8 which is similar to our clinical results [10].

The present study has some limitations. Although the cantilever experimental setup is recognized as physiological when it comes to imitating load to the arm, it is considered unfavorable for evaluating screw pullout. Some studies observed frequent failure next to the plate or bending of the plate. Also, human motion is more complex than in the uniaxial test setup we used. Multidirectional test setups, such as robotic systems, which are already established for hip and knee testings [38–41], should be implemented and used for further investigations. Furthermore, heterogeneous fracture groups were compared. In our clinical routine a shortened osteosynthesis (2 screws per fracture side) is only used for simple fractures, which we compared to standard osteosynthesis techniques (3 screws per fracture side). The clinical follow-up contains only a small clinical case series and a comparison group was not available. Also, the length of the skin incision was not measured in all patients, therefore we cannot prove a reduction of the skin incision length in our study, although it was observed.

## Conclusions

For certain fracture morphologies such as a non-fragmented and well interlocking 2-part clavicle shaft fracture, minimally invasive plate osteosynthesis using 2 screws per fracture side (e.g. 5-hole LCP) might offer sufficient biomechanical stability, comparable fusion rates and better preservation of the soft tissue. However, the clinical relevance of biomechanical studies might be arguable. Also, bicortical screw fixation and exact positioning is indispensable, otherwise the risk of osteosynthesis failure increases and a larger plate (e.g. 7-hole LCP) has to be used.

## Supplementary Information

**Additional file 1: Fig 8. **radiological example of a failed 5-hole plate osteosynthesis (e and f) after treatment of a 2-part clavicle shaft fracture (15.2A according to AO classification) (a and b) with a 5-hole reconstruction plate and 2 non-locking screws on each side of the fracture (c and d) and radiological result 6 weeks after revision surgery with conversion to a 6-hole LCP (g and h).

## Data Availability

The datasets generated and/or analysed during the current study are available from the corresponding author on reasonable request.

## Abbreviations

AO: German for ‘Arbeitsgemeinschaft für Osteosynthesefragen ‘
CS: Constant Score
CT: Computer tomography
DASH: Disabilities of Arm, Shoulder and Hand
e.g.: Latin: ‘exempli gratia’; English: for example
Fig.: Figure
LCP: Locking compression plate
max.: Maximum
min.: Minimum
mm: Millimeter
N: Newton
SD: Standard deviation
vs.: Versus
