# Supplementary figures and images for: Biomechanical and clinical evaluation of minimal invasive plate osteosynthesis for two-part clavicle shaft fractures
Source: BMC Musculoskelet Disord. 2023 Jul 25;24:612. doi: 10.1186/s12891-023-06699-x (PMC10369786; doi:10.1186/s12891-023-06699-x)

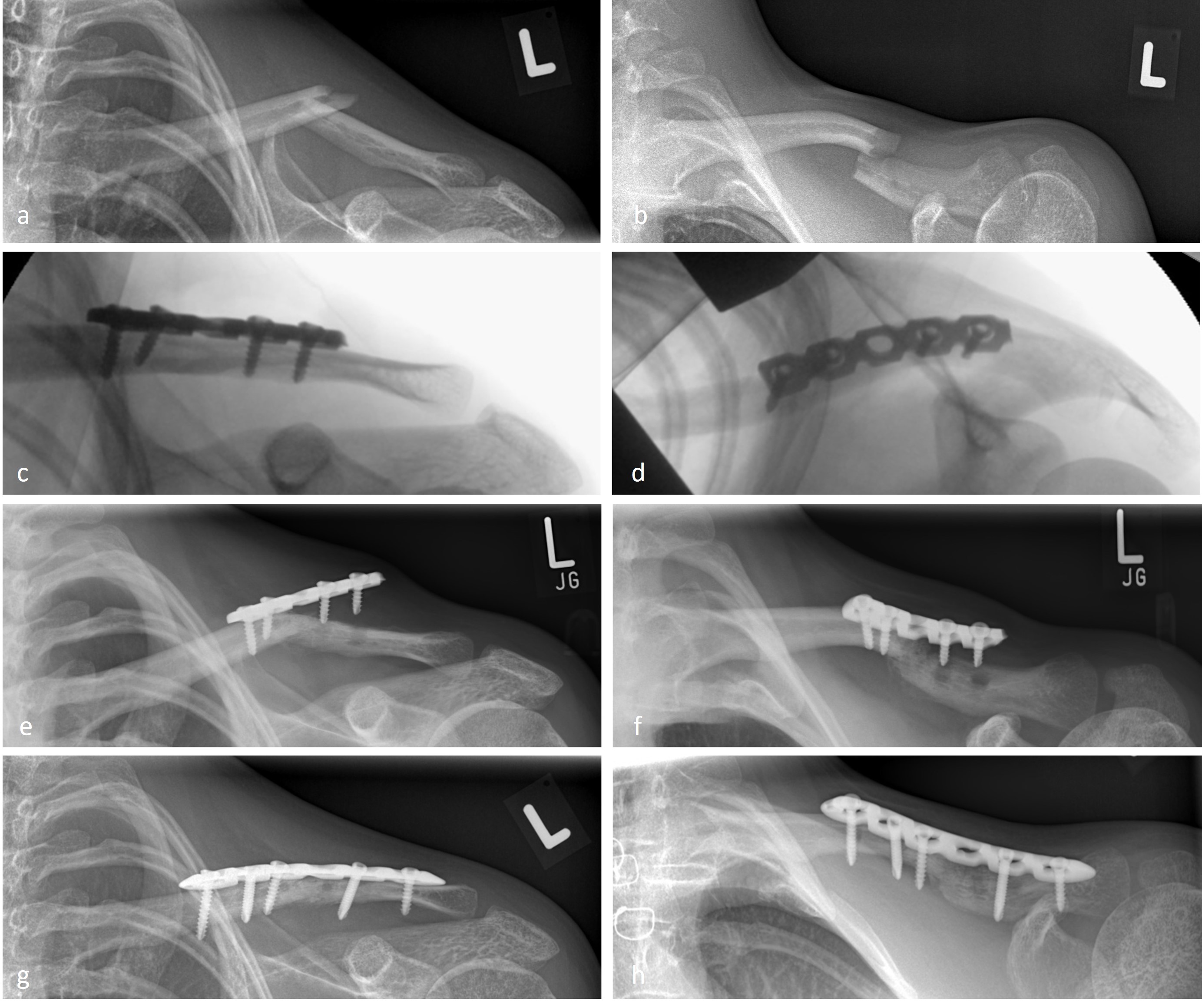

Supplement: Supplementary file 1 — Additional file 1: Fig 8. radiological example of a failed 5-hole plate osteosynthesis (e and f) after treatment of a 2-part clavicle shaft fracture (15.2A according to AO classification) (a and b) with a 5-hole reconstruction plate and 2 non-locking screws on each side of the fracture (c and d) and radiological result 6 weeks after revision surgery with conversion to a 6-hole LCP (g and h). [file 12891_2023_6699_MOESM1_ESM.jpg]
